# Developmental Effects of (Pre-)Gestational Diabetes on Offspring: Systematic Screening Using Omics Approaches

**DOI:** 10.3390/genes12121991

**Published:** 2021-12-15

**Authors:** Bachuki Shashikadze, Florian Flenkenthaler, Jan B. Stöckl, Libera Valla, Simone Renner, Elisabeth Kemter, Eckhard Wolf, Thomas Fröhlich

**Affiliations:** 1 Laboratory for Functional Genome Analysis (LAFUGA), Gene Center, LMU Munich, 81377 Munich, Germany; shashikadze@genzentrum.lmu.de (B.S.); flenkenthaler@genzentrum.lmu.de (F.F.); stoeckl@genzentrum.lmu.de (J.B.S.); 2 Chair for Molecular Animal Breeding and Biotechnology, Gene Center and Department of Veterinary Sciences, LMU Munich, 81377 Munich, Germany; Libera.Valla@gen.vetmed.uni-muenchen.de (L.V.); Simone.Renner@lmu.de (S.R.); Kemter@genzentrum.lmu.de (E.K.); 3 Center for Innovative Medical Models (CiMM), LMU Munich, 85764 Oberschleißheim, Germany; 4 German Center for Diabetes Research (DZD), 85764 Neuherberg, Germany

**Keywords:** DOHaD (developmental origins of health and disease), gestational diabetes mellitus (GDM), pregestational diabetes mellitus (PGDM), Omics

## Abstract

Worldwide, gestational diabetes affects 2–25% of pregnancies. Due to related disturbances of the maternal metabolism during the periconceptional period and pregnancy, children bear an increased risk for future diseases. It is well known that an aberrant intrauterine environment caused by elevated maternal glucose levels is related to elevated risks for increased birth weights and metabolic disorders in later life, such as obesity or type 2 diabetes. The complexity of disturbances induced by maternal diabetes, with multiple underlying mechanisms, makes early diagnosis or prevention a challenging task. Omics technologies allowing holistic quantification of several classes of molecules from biological fluids, cells, or tissues are powerful tools to systematically investigate the effects of maternal diabetes on the offspring in an unbiased manner. Differentially abundant molecules or distinct molecular profiles may serve as diagnostic biomarkers, which may also support the development of preventive and therapeutic strategies. In this review, we summarize key findings from state-of-the-art Omics studies addressing the impact of maternal diabetes on offspring health.

## 1. (Pre-)Gestational Diabetes Mellitus and Omics: A Brief Introduction

Pregnancy is a dynamic state associated with major metabolic adaptations [1,2], being crucial for fetal development [3], delivery, and breastfeeding [4]. The primary source of energy for the fetus is maternal glucose [5]. An increased rate of hepatic glucose production [6], combined with insulin resistance (IR), are important mechanisms adopted by the mother to meet the high demand for glucose. The development of IR, which has diabetogenic effects during pregnancy [7], is an evolutionary mechanism to minimize maternal glucose utilization and to ensure an adequate supply for the growing fetus [8]. In addition, during normal pregnancy, as a response to elevated glucose production and decreased insulin sensitivity (equivalent to IR), β cells need to undergo changes to further elevate insulin synthesis and to maintain a normoglycemic state [9]. Furthermore, insulin in concert with placenta-derived hormones [10] reprograms the metabolism of lipids [11], leading to the accumulation of maternal fat in early and mid-pregnancy and promoting fat utilization at a later stage.

The inability to compensate for the increased demand for insulin during pregnancy underlies the pathophysiological mechanisms of gestational diabetes mellitus (GDM) [12]. GDM is one of the most common complications of pregnancy, with a prevalence ranging from 2% to 25% depending on the used diagnostic criteria and the studied population [13,14,15,16]. GDM is defined as glucose intolerance, firstly diagnosed during pregnancy [17]. Obesity, a family history of diabetes, and previous GDM pregnancies are among the major risk factors for developing GDM [18]. In the case of GDM, maternal glucose tolerance usually normalizes shortly after pregnancy but leads to a substantially increased risk of developing type 2 diabetes in later life [19]. Apart from GDM, pre-existing, poorly controlled diabetes can also lead to maternal hyperglycemia. Elevated maternal glucose can penetrate through the placenta and reach the fetus, while insulin cannot [20]. The arising hyperglycemia can lead to insulin overproduction (hyperinsulinemia) in the fetus. This phenomenon was first described by Jorgen Pedersen and is known as Pedersen’s hypothesis [21]. Such an aberrant intrauterine environment induced by maternal diabetes is related to an increased risk for complications both for the mother and the offspring (Table 1).

The broad majority of diseases and metabolic disorders are associated with imbalances in the complex network of biological molecules necessary to accomplish a particular biological function. High-throughput Omics technologies analyzing complex mixtures of biological molecules, in combination with advanced data mining and rigorous statistical tools, have reshaped biomedical research. In the last few decades, the application of Omics studies on different molecular levels (e.g., genomics, epigenomics, transcriptomics, proteomics, and metabolomics) successfully deciphered the complex nature of various diseases [29]. Omics research in maternal diabetes and associated offspring health is still in an exploratory phase (i.e., screening for novel biomarkers, revealing dysregulated biological pathways). The screening and diagnostic methods for GDM are mainly based on glucose metabolism (e.g., fasting plasma glucose (FPG), oral glucose tolerance test (OGTT), glycated hemoglobin, etc.). However, currently, there is no broad consensus on appropriate screening/diagnostic tests for GDM (discussed in [30]). Additionally, there are no effective strategies to prevent health complications to offspring due to the lack of systematic insight into maternal diabetes-associated molecular derangements.

**Figure 1:**
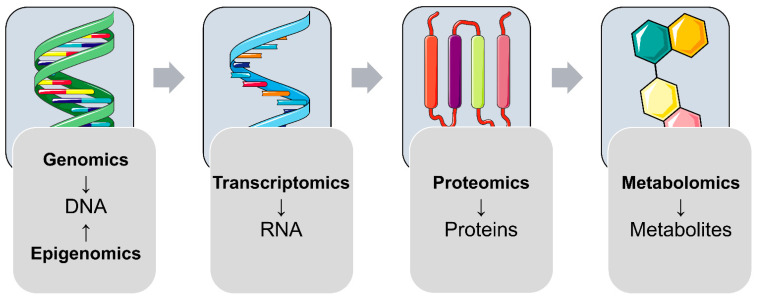
Overview of Omics fields addressing different classes of molecules. Interactions of the different classes of molecules can be addressed using more than one Omics technique in a so-called multi-Omics approach.

With the “central dogma of molecular biology” in mind that the genetic information of a biological system is encoded in the DNA and is transcribed to RNAs, which are translated to functional proteins, controlling an organism’s metabolism (Figure 1), this review is focused on recent findings of (epi-)genomics, transcriptomics, proteomics, and metabolomics studies, addressing molecular changes in offspring after exposure to (pre-)gestational diabetes. Furthermore, studies from other growing Omics fields, such as microbiomics and nutriomics, will be discussed. In this review, we mainly focus on studies reflecting molecular changes observed at birth (i.e., cord blood studies, fetal-side placenta, and tissues from different animal models) and in later life.

## 2. Animal Models Are Valuable for Studying Effects of Maternal Diabetes on Offspring

**Figure 2:**
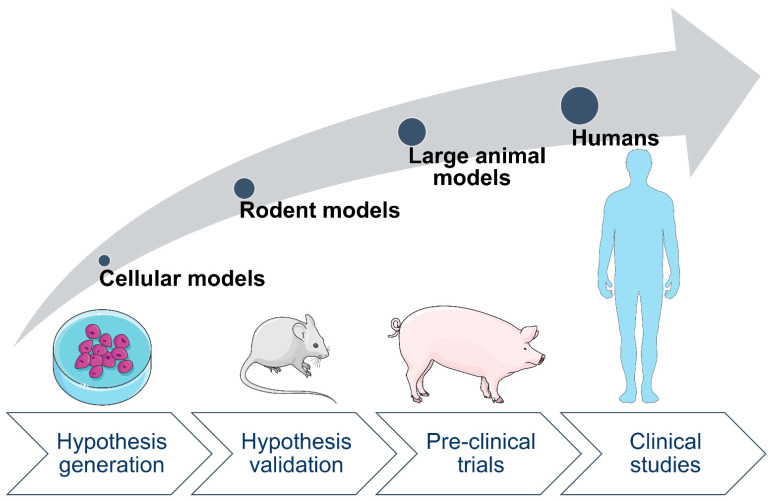
Overview of the different model systems used for translational research. Easy to handle and reproducible cellular models are useful for deciphering molecular disease mechanisms, which can be validated in animal models. Large animal models better mimicking human diseases are valuable to fill the gap between proof-of-concept studies and clinical trials.

Studies on the negative effects of maternal diabetes on the health of human offspring show several limitations due to the number of co-occurring factors, such as the person’s lifestyle and medical history. For example, most of the human studies include cases of maternal diabetes accompanied by other metabolic disorders, which makes it difficult to differentiate the consequences of diabetes from those of comorbidities. Therefore, animal models living under tightly controlled laboratory environments with the option of standardized tissue sampling [31] are necessary. So far, a variety of animal models have been generated to study GDM [32]. For instance, rodent models for GDM, generated by the usage of chemicals leading to β cell loss, are widely used. However, rodent models frequently lack clinical relevance due to fundamental physiological differences from humans. Clinically more relevant, large animal models have the potential to bridge the gap between proof-of-concept studies and clinical trials (Figure 2). Non-human primate models have been used to study the developmental programming of diabetes and obesity [33]. Pigs are also attractive animal models due to their similarities with humans in anatomy and metabolism [34]. For diabetes, specific characteristics are particularly relevant (e.g., size and distribution of β cells, similarity in insulin structure) that make the pig a valuable model for human glucose metabolism [35,36]. Importantly, despite structural differences between porcine and the human placenta (epitheliochorial vs. hemochorial), the transfer of glucose, amino acids, and partially fatty acids towards the fetus take place in both species [37].

## 3. Common Tissues and Biofluids for Studying Effects of Maternal Diabetes on Offspring

The use of Omics technologies on human samples and samples from clinically relevant animal models, which enable the detection of molecular changes in pathological conditions, represents a promising strategy to study the effects of maternal diabetes on offspring future health at a mechanistic level. Depending on the experimental aim, biological samples can be collected from the mother (plasma, urine, vaginal fluids, milk, placenta, and hair) or the fetus/newborn (amniotic fluid, umbilical cord blood, plasma, urine, meconium, and saliva, etc.) [38]. Due to ethical reasons, human samples can only be collected by non or minimally invasive procedures. Therefore, cord blood is frequently used to explore diabetes-induced molecular changes in human neonates. Longitudinal studies exploring the postpartum effect of maternal diabetes often use the offspring’s peripheral blood. These studies will be extensively discussed in this review. Furthermore, molecular changes in the placenta might be reflective of disorders during fetal development. However, as the placenta is only available at birth, it is unclear to what extent alterations observed at delivery resemble those in utero during fetal development [39]. Furthermore, not every study specifies if the maternal or the fetal side of the placenta was analyzed. Therefore, this review focuses on those studies that specifically analyzed the fetal side of the placenta (also reviewed in [40,41]). To verify whether the findings from cord blood or placenta studies reflect molecular derangements of different organs, animal models, which facilitate the molecular profiling of various tissues, are necessary. Therefore, studies from human offspring and animal models will be discussed side-by-side in this review.

## 4. (Epi)Genetic Factors Affecting Offspring Outcomes after Exposure to Maternal Diabetes

An organism’s complete set of DNA is referred to as a genome. Genomics, which is the oldest and most established of the Omics disciplines, uses various methods, including DNA sequencing combined with bioinformatics, to study the structure and function of genomes [42]. Genome-wide association studies (GWAS) are powerful approaches to associate genetic variation with traits such as particular disease states [43].

**Figure 3:**
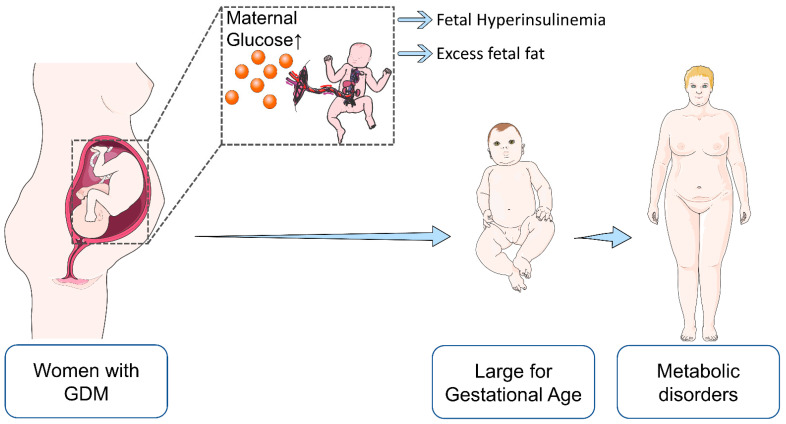
Maternal diabetes predisposes offspring to future metabolic disorders. In response to elevated maternal glucose supply, the fetus increases insulin secretion, resulting in hyperinsulinemia, increased body fat, and subsequently higher birth weight (LGA). The latter is a risk factor for future metabolic syndrome, type 2 diabetes and obesity.

In extensive population-based studies, elevated maternal glucose levels were associated with “large for gestational age” (LGA) fetuses, which is particularly relevant since high birth weight is among the risk factors of future metabolic disorders, including obesity [44,45] (Figure 3). In the context of GDM, insulin and glucose, together with several adipokines (leptin, adiponectin, and others), are thought to be involved in imbalanced fetal growth [46]. Interestingly, Hughes et al. reported that not only maternal glucose but also the fetal genotype has an effect on birth weight. The authors generated a fetal genetic score using birth weight-associated single nucleotide polymorphisms (SNPs) and investigated their associations with the offspring birth weights at varying levels of maternal fasting plasma glucose (FPG). For FPG levels, data from “The Exeter Family Study of Childhood Health” (EFSOCH) [47] and “The Hyperglycemia and Adverse Pregnancy Outcome” (HAPO) [48] study were used. Interestingly, no association between the fetal genetic score and cord blood insulin or C-peptide was found. The fetal genetic score influenced birth weight independently of maternal FPG and impacted growth at all levels of maternal glycemia. The authors concluded that fetal genetics has a major impact on fetal growth and mainly acts through mechanisms independent of FPG levels [49].

Apart from mutations or genetic variations, covalent modifications of DNA and histones are major regulators of gene transcription and are therefore determinants of cell fate [50]. These modifications, without accompanying changes of the nucleotide sequence, are collectively referred to as an epigenome and are the focus of the epigenomics field. The most extensively studied epigenetic modification is DNA methylation, which is known to be responsive to environmental stimuli [51]. DNA methylation is not limited to but is generally associated with repression of gene expression (gene silencing) [52,53]. Genome-wide DNA methylation variations induced by GDM are supposed to have an important role in metabolic disease programming in offspring [54].

Epigenomic changes in offspring induced by maternal diabetes have been the focus of multiple research projects (Table 2) and are discussed in various review articles [22,55,56,57,58,59]. Therefore, only major findings from selected studies will be discussed here. Although a variety of these studies were performed with small sample sizes and limited statistical power, several of the obtained results suggest that elevated maternal glucose during pregnancy is associated with DNA methylation at the cytosine–guanine (CpG) dinucleotide sites in genes related to metabolic functions within the offspring’s genome. These epigenetic alterations are further supposed to result in a predisposition for metabolic, neurodevelopmental, and immune-related disorders.

For example, a significant influence of elevated maternal glucose on the epigenetic pattern of the offspring’s leptin (*LEP*) gene was demonstrated. While Lesseur et al. found a 2.5 % increased methylation level of *LEP* in the fetal placenta portion in GDM subjects [60], other studies revealed lower methylation levels of *LEP* in the fetal-side placenta [61] and in the cord blood [62], which was associated with increased cord blood leptin levels. In a study by Côté et al., more differentially methylated genes were found in the fetal-side placenta from hyperglycemic pregnancies. Interestingly, altered methylation of the peroxisome proliferator-activated receptor-γ co-activator 1 α (*PPARGC1A*) gene mediated the association between maternal hyperglycemia and the cord blood leptin levels [63]. A key function of leptin is to regulate energy balance [64,65], and in the “Project Viva” prospective cohort study, leptin levels in the cord blood were associated with elevated adiposity rates at the age of 3 years [66]. Therefore, altered DNA methylation at the *LEP* locus might contribute to the increased risk of obesity in offspring born to hyperglycemic mothers.

Epigenetic variation in the context of maternal diabetes was also detected at other adipokine loci. For instance, Bouchard et al. found that elevated maternal glucose levels during the second trimester of pregnancy were correlated with lower methylation levels of the adiponectin (*ADIPOQ*) gene promoter in the fetal placenta portion. The authors hypothesized that this could be one of the mechanisms involved in the fetal programming of metabolic disorders [67]. Houde et al. found reduced methylation of both *LEP* and *ADIPOQ* in the fetal-side placenta with increased maternal blood glucose concentrations. The authors concluded that maternal hyperglycemia has similar effects on both genes [68]. Taken together, it is conceivable that maternal diabetes influences epigenetic marks of adipokine genes, which predisposes offspring to future metabolic disorders.

In line with the role of the placenta in fetal–maternal communication and nutrient transfer, several studies investigated epigenetic changes of genes encoding transporter proteins. Houde et al. found in GDM pregnancies a reduced methylation level of the lipoprotein lipase (*LPL*) gene in the fetal part of the placenta. *LPL* plays a role in the transfer of fatty acids to the fetus. Furthermore, epivariation of *LPL* was associated with cord blood lipid levels. The authors concluded that the methylation level at a specific *LPL* CpG locus might influence placental lipid flux and lipid metabolism in the offspring [69]. Another study showed that epivariation near the *LPL* locus in the fetal-side placenta correlates with anthropometric characteristics (i.e., birth weight, mid-childhood weight, fat mass) of children at the age of 5 years [70].

In addition to altered methylation of the genes coding for lipid transporters, reduced methylation of the serotonin transporter (*SLC6A4*) gene was found in the fetal side of the placenta from GDM pregnancies [71]. Serotonin is a multifunctional signaling molecule associated with mental health conditions, including autism spectrum disorder [72]. Interestingly, a recent meta-analysis suggests a possible association of maternal GDM with an autism spectrum disorder in offspring [73].

Multiple studies investigated epigenetic changes in cord blood in the context of maternal diabetes. For instance, Howe et al. [13] analyzed the association between maternal GDM and cord blood DNA methylation in the pregnancy and childhood epigenetics (PACE) consortium [74] and identified two hypomethylated regions; one in the gene body of *CYP2E1* and another one in the *OR2L13* promoter. These genes are associated with type 1/2 diabetes and with an autism spectrum disorder, respectively [13]. As a potential mechanism for maternal diabetes-induced autism spectrum disorder, Wang et al. found that maternal hyperglycemia suppressed superoxide dismutase 2 (*Sod2*) expression in the amygdala of rat offspring, resulting in autism-like behavior. The authors further found that the *Sod2* suppression may result from oxidative stress-mediated histone methylation and the subsequent dissociation of the transcription factor early growth response 1 (EGR1) from the *Sod2* promoter in neurons [75].

A genome-wide comparative methylome analysis of cord blood samples from offspring of GDM and normal pregnancies detected differential methylation patterns of genes mainly involved in pathways related to type 1 diabetes mellitus, immune system/major histocompatibility complex (MHC), and neuron development [76].

Furthermore, epigenetic dysregulation of the mesoderm specific transcript (*MEST*) gene was suggested as a possible risk factor for obesity in offspring. El Hajj et al. observed reduced DNA methylation levels of *MEST* in the cord blood of GDM subjects. In addition, the authors detected significantly lower DNA methylation of *MEST* in the blood of morbidly obese adults [77].

Various studies focused on epigenomic programming effects being present years after birth in offspring born to diabetic mothers. For instance, Shiau et al. investigated the association between prenatal GDM exposure and offspring DNA methylation at ages 3–10 years postpartum and found signs of accelerated epigenetic aging associated with cardiometabolic risk factors [78]. The association between the exposure to maternal diabetes in utero and the risk of cardiometabolic disorders in children (aged 8–12 years) was confirmed by another study performing genome-wide methylation analyses of peripheral blood mononuclear cells. Several genes, known to be associated with cardiometabolic traits, were found to be differentially methylated [79]. Yang et al. [80] performed DNA methylation analyses of blood samples from the Exploring Perinatal Outcomes among Children (EPOCH) cohort [81] (average age of investigated subjects: 10.5 years) and found in offspring exposed to GDM several differentially methylated regions, including loci linked to adiposity. In particular, methylation of the *SH3PXD2A* gene was significantly associated with multiple adiposity-related outcomes, including body mass index (BMI), waist circumference, as well as blood leptin levels [80]. Hjort et al. determined DNA methylation profiles in the peripheral blood of GDM-exposed and unexposed children aged between 9–16 years and validated potentially GDM-associated, differentially methylated CpGs in a larger replication cohort. Ingenuity pathway analysis (IPA) analysis showed enrichment of various functional networks, with lipid metabolism ranking highest. The authors further discussed the association of the identified differentially methylated genes with type 2 diabetes, obesity, diabetic nephropathy, or coronary heart disease, as found in previous reports [82].

**Table 2:** Summary of selected human studies linking maternal diabetes with the (epi-)genome profiles of offspring.

Maternal Characteristics	Bio-Specimen	Major Findings in Offspring	Reference
GDM	Fetal-side placenta	Reduced methylation level of *LEP*, contributing to cord blood leptin level regulation	[61]
GDM	Fetal-side placenta	Increased *LEP* methylation	[60]
GDM	Fetal-side placenta	Altered methylation of *PPARGC1A* mediating the association between maternal hyperglycemia and cord blood leptin levels	[63]
GDM	Fetal-side placenta	DNA methylation profile of *ADIPOQ* was associated with maternal glucose status	[67]
GDM	Fetal-side placenta	Reduced *LPL* methylation	[69]
GDM	Fetal-side placenta	Epivariation near the *LPL* locus correlated with anthropometric parameters (birth weight, mid-childhood weight, fat mass) of children at age 5 years	[70]
GDM	Fetal-side placenta	Reduced *SLC6A4* DNA methylation	[71]
GDM	Cord blood and chorionic villi	Decreased *MEST* methylation	[77]
GDM	Cord blood	Altered methylation of the *OR2L13* promoter (a gene associated with autism spectrum disorder) and of the gene body of *CYP2E1* (which is upregulated in type 1 and type 2 diabetes)	[13]
GDM	Cord blood	Differentially methylated genes associated with type 1 diabetes mellitus, immune MHC, and neuron development	[76]
GDM	Cord blood	Decreased *LEP* methylation; association with increased cord blood leptin levels	[62]
GDM	Peripheral blood	Differentially methylated genes associated with type 2 diabetes, obesity, diabetic nephropathy or coronary heart disease	[82]
GDM	Peripheral blood mononuclear cells	Differential methylation of several genes known to be associated with cardiometabolic traits;	[79]
GDM	Peripheral blood	Accelerated epigenetic aging associated with cardiometabolic risk factors	[78]
GDM	Peripheral blood	Methylation of *SH3PXD2A* was associated with multiple adiposity-related outcomes, including BMI, waist circumference, and circulating leptin levels	[80]

## 5. Transcriptomic Changes in Offspring after Exposure to Maternal Diabetes

Ribonucleic acid (RNA) transcripts can have various biological functions, such as carrying genetic information from the genome as well as regulating gene expression, both essential for an organism’s survival [83]. The entirety of all coding and non-coding RNA transcripts of a cell or tissue at a given timepoint is referred to as a transcriptome [84]. Transcriptomics technologies can be used to study an organism’s transcriptome qualitatively (e.g., checking the presence of a transcript and spotting new splice variants) and quantitatively (determination of levels of given RNA species) [50]. Unlike DNA, which is generally static, RNA levels are sensitive to epigenetic regulation as well as to environmental stimuli (e.g., diseases) [85]. Two widely used techniques in the field of transcriptomics are DNA microarrays and the more powerful RNA sequencing (RNA-seq). While the former quantifies a set of predetermined sequences by hybridization with probes spotted on a solid support, RNA-seq mostly uses next-generation sequencing (NGS) and is able to capture whole transcriptomes [86] in a highly effective and sensitive way. Since gene expression is mediated by messenger RNAs (mRNAs), high-throughput transcriptome studies mostly focus on this type of transcript.

The impact of maternal diabetes on offspring gene expression was the focus of several studies (Table 3). To study the effects of chronic hyperglycemia on the fetal vascular cell transcriptome, Ambra et al. used Affymetrix microarrays to analyze human umbilical vein endothelial cells (HUVEC) obtained from GDM women at delivery. Several genes coding for growth factors linked to insulin sensing and to the extracellular matrix were upregulated in GDM-HUVEC cells [87]. Furthermore, Koskinen et al. used microarrays to determine gene expression profiles in umbilical cord tissues from neonates born to type 1 diabetic mothers, compared to neonates born to a healthy control group. The authors found that maternal diabetes had a major effect on the expression of genes involved in vascular development, vessel wall integrity, and vascular function. Additionally, the authors hypothesized that the observed alterations in cords might similarly occur in the developmental regulation of various tissues in the offspring of diabetic mothers [88].

Other studies used animal models to reveal the effects of maternal diabetes on transcriptome profiles in the offspring’s pancreas and liver, two major organs controlling blood sugar levels. Casasnovas et al. utilized an infusion model for localized fetal hyperglycemia in rats. Using a vascular catheter, glucose delivery was targeted to fetuses residing in the left uterine horn, allowing the use of fetuses in the right uterine horn as genetically similar controls with normal glucose levels. RNA-seq of pancreatic islets from gestational day 22 (GD22) fetuses detected 87 differentially expressed genes (DEGs) in hyperglycemia-exposed offspring, which were associated with diabetes mellitus as well as inflammation and cell-death pathways [89]. In a further study, Inoguchi et al. investigated the liver gene expression profiles of offspring of poorly controlled diabetic female mice generated by streptozotocin (STZ) administration. Pathway enrichment analysis showed “FOXO signaling pathway” and “PPAR signaling pathway” to be enriched in the set of DEGs of male offspring, while genes related to “AMPK signaling pathway” and “Fatty acid metabolism pathway” as well as “PPAR signaling pathway” were overrepresented in the set of DEGs from the female offspring. A key finding of this study was an increased activation of the forkhead box protein O1 (*Foxo1*) gene in the liver of the male offspring, associated with increased FOXO1 protein levels and a decreased phosphorylation at Ser256, inhibiting its activity. Moreover, in male but not female offspring, the transcript levels of two gluconeogenic genes, glucose 6-phosphatase catalytic subunit (*G6pc*) and phosphoenolpyruvate carboxykinase 1 (*Pck1*), were upregulated. The authors suggested that dysregulation of FOXO1 target genes in the liver may contribute to increased gluconeogenesis in male offspring. However, these changes were not pronounced in female offspring [90]. Interestingly, decreased phosphorylation levels of FOXO1 but an increased total of FOXO1 protein levels with increased abundance of PCK1 and other gluconeogenic enzymes were also detected in the livers of female transgenic pigs expressing mutant insulin C94Y, a model of insulin-deficient diabetes mellitus. Using transcriptomics, proteomics, and metabolomics analysis, the authors were able to support the hypothesis that increased gluconeogenesis in insulin deficiency is associated with elevated levels of retinol dehydrogenase 16 (RDH16) and its metabolic product all-trans retinoic acid that stimulates the expression of *PCK1* [91].

Several studies indicated that, apart from the pancreas and liver, other human organs, such as the brain, are affected by maternal diabetes. For example, an increased risk of future weight gain or obesity in children exposed to maternal diabetes was associated with hypothalamic transcriptome alterations [92]. Moreover, the brain is particularly relevant since maternal diabetes is supposed to predispose offspring to neurodevelopmental and cognitive disorders. The association between maternal diabetes and cognitive impairments in infants is underpinned by the systematic review from Robles et al. [93]. Addressing the effects of GDM on the brain transcriptome, Aviel-Shekler et al. investigated a mouse model of diabetes based on STZ administration to pregnant mice. RNA-seq analysis of brains from male offspring detected a dysregulation of only nine genes in the frontal cortex, which were related to forebrain development. While no significant change of gene expression was observed in striatum, weighted correlation network analysis (WGCNA) revealed dysregulation of neurodevelopment- and immune-related genes [94]. Furthermore, Money et al. investigated in mice the impact of diet-induced maternal diabetes alone or in combination with maternal immune activation (MIA) on the developing brain of offspring. Interestingly, each condition alone resulted in altered expression profiles of genes related to inflammatory and neurodevelopmental processes, which was even worsened in the combination of GDM and MIA. Interestingly, GDM increased the expression of vascular endothelial growth factor A (*Vegfa*) mRNA, which is known to be associated with hypoxic conditions. The authors suggested that the altered hypoxia-related signature might be a consequence of an increased oxygen requirement due to an elevated metabolic demand associated with maternal hyperglycemia [95]. The main regulator of responses to a hypoxic environment is hypoxia-inducible factor 1 (HIF1) [96]. In line with this, Cerychova et al. investigated in a mouse model combinatorial effects of maternal diabetes and haploinsufficiency of *Hif1a* on the heart’s left ventricles (LV) of offspring. The authors found that the combination of maternal diabetes and *Hif1a* haploinsufficiency results in significant metabolic, structural, and functional changes in the LV myocardium. Additionally, RNA-seq analysis revealed alterations of transcripts associated with metabolic processes, including two genes that are known to be HIF1A targets: *Cd36* and lactate dehydrogenase A (*Ldha*). The authors concluded that HIF1A deficiency and maternal diabetes exposure increase the predisposition to cardiac dysfunction in offspring [97]. These findings obtained in a mouse model may be of translational importance because a predisposition for cardiovascular disorders (CVD) was demonstrated in a recent population-based human cohort study with 40 years of follow-up. The authors demonstrated that children born to a mother with diabetes have increased rates of early-onset CVD [98]. In also addressing the impact of maternal diabetes on the heart transcriptome in offspring, Preston et al. used microarray gene expression profiling of heart samples from newborn offspring of diabetic rats, rats exposed to a high-fat diet, or the combination of both. Diabetes in female rats was induced via the administration of STZ during pregnancy. While the combination of diabetes and a high-fat diet resulted in more pronounced changes, diabetes alone induced only a few changes. Among the differentially abundant transcripts were α hemoglobin stabilizing protein (*Ahsp*) and Kell metallo-endopeptidase (*Kel*), both encoding proteins highly expressed in red blood cells. The authors assumed that the higher levels of *Ahsp* and *Kel* mRNA were due to a higher number of residual red cells in the myocardial vasculature, and they put this into the context of neonatal polycythemia, which is a common complication of maternal diabetes [99].

**Table 3:** Summary of selected studies linking maternal diabetes with the transcriptome profiles of offspring.

Species	Maternal Characteristics	Bio-Specimen	Major Findings in Offspring	Reference
Human	GDM	HUVEC	Increased mRNA levels of genes coding for growth factors linked to insulin sensing and to the extracellular matrix	[87]
Human	Type 1 diabetes	Umbilical cord	Altered expression of genes involved in vascular development, vessel wall integrity, and vascular function	[88]
Rat	STZ-induced diabetes	Heart	Altered expression of *Ahsp* and *Kel*; possible relation to polycythemia	[99]
Mouse	STZ-induced diabetes	Heart	Altered expression of *Cd36* and *Ldha* induced by maternal diabetes plus haploinsufficiency of *Hif1a*	[97]
Mouse	STZ-induced diabetes	Brain	Dysregulation of genes in frontal cortex related to forebrain development; dysregulation of neurodevelopment and immune-related genes in the striatum	[94]
Mouse	Diet-induced diabetes	Brain	Altered expression of genes related to inflammatory and neurodevelopmental processes	[95]
Rat	Infusion model of localized hyperglycemia	Islets	Dysregulation of genes associated to diabetes mellitus, inflammation and cell-death pathways	[89]
Mouse	STZ-induced diabetes	Liver	Differential expression of genes related to “FOXO signaling pathway” and “PPAR signaling pathway” in male offspring, and of genes related to “AMPK signaling pathway”, “fatty acid metabolism pathway”, and “PPAR signaling pathway” in female offspring	[90]

## 6. Proteomic Changes in Offspring after Exposure to Maternal Diabetes

The proteome is the complete set of proteins expressed by a cell, tissue, or organism at a given state. Proteins are the primary functional actors of the cell, performing diverse functions, such as catalyzing chemical reactions, facilitating cellular transport, mediating signaling and many other tasks necessary for living organisms. The systematic large-scale identification and quantification of proteins and the analysis of their post-translational modification are called proteomics. Even though NGS-based transcriptomics is a very powerful technique with outstanding analytical depth, comparisons between transcriptomic and proteomic data indicate that only about 40% of variations within proteomes can be explained by altered transcript levels [100]. This clearly demonstrates that transcriptome data are not sufficient to predict protein levels [101]. The need to overcome this limitation led to the rapid development of sophisticated proteomics techniques, of which mass spectrometry has emerged as the most effective and sensitive technology to quantify proteins in complex biological mixtures. Discovery proteomics (also known as shotgun proteomics) is routinely used to effectively characterize the proteome of interest, whereas targeted proteomics focuses on a predefined set of proteins, allowing a more accurate quantification with a high dynamic range.

Although data at the proteome level are essential to understanding disease-related biochemical networks, so far, mainly four high-throughput studies have investigated the impact of maternal diabetes on offspring proteomes (Table 4). Kopylov et al. compared cord blood samples from patients with different types of diabetes mellitus (GDM, type 1 diabetes, type 2 diabetes) who delivered either healthy newborns or newborns with fetopathy complications. The most altered proteins in the cord blood across the groups were apolipoprotein M (APOM), ceruloplasmin (CP), plasminogen (PLG), angiotensinogen (AGT), kininogen-1 (KNG1), apolipoprotein A-I (APOA1), α-1-acid glycoprotein 2 (ORM2), serotransferrin (TF), histidine-rich glycoprotein (HRG), apolipoprotein D (APOD), and lumican (LUM). Bioinformatics analysis revealed processes such as inflammation, extracellular matrix remodeling, and lipid metabolism, etc., possibly altered due to maternal diabetes [102]. Altered lipid metabolism and possible relation to GDM-induced macrosomia was also shown in the study by Miao et al., who analyzed the cord blood of GDM patients whose offspring showed obesity at ages 6–7 years. In total, 318 proteins were identified by liquid chromatography with tandem mass spectrometry (LC-MS/MS), of which 36 were differentially abundant. The three randomly chosen proteins, rho guanine nucleotide exchange factor 11 (ARHGEF11), phospholipid transfer protein (PLTP), and lecithin-cholesterol acyltransferase (LCAT), were further validated by western blot (WB) and were consistent with LC-MS/MS results. The authors suggested a close relation of these proteins to abnormalities in glucose and lipid metabolism, while ARHGEF11 is known to influence embryo development. Furthermore, IPA revealed a connection of the differentially abundant proteins to adenocarcinoma. The authors concluded that GDM offspring might have an increased risk of adenocarcinoma, which has to be confirmed by follow-up studies [103]. Similar to PLTP and LCAT, another protein involved in lipoprotein metabolism, the cholesteryl ester transfer protein (CEPT), was found differentially abundant in a study by Liao et al. [104] comparing umbilical venous plasma samples from offspring of GDM patients and control subjects. Out of 780 identified proteins, 98 proteins were found to be differentially abundant in umbilical venous plasma of GDM patients compared to controls. Six of these proteins were also consistently regulated in maternal peripheral plasma samples, including CEPT and apolipoprotein M (APOM) that are known to be GDM-related. Notably, in line with the above-mentioned study by Kopylov et al. [102], APOM was one of the most elevated proteins in the cord blood as a response to maternal diabetes. Furthermore, CEPT concentration in umbilical venous plasma was found to be correlated with the low-density lipoprotein (LDL) levels. Alterations of CEPT abundance were confirmed using an enzyme-linked immunosorbent assay (ELISA) and remained significant after adjustment for age and neonatal gender. Additionally, bioinformatics-based IPA analysis predicted the follicle-stimulating hormone (FSH) as an upstream regulator of the detected differentially abundant proteins [104]. FSH is essential for normal ovarian follicular maturation [105]. In line with this, in the study by Clark et al., healthy follicles were decreased in ovaries from mice offspring after in utero GDM exposure and dietary stress during adulthood. In this study, aiming to investigate the role of GDM on the developmental origins of ovarian disorder, GDM was induced by feeding female mice with a high-fat, high-sucrose diet (HFHS) one week prior to mating and for the duration of gestation. Offspring of HFHS fed mice and control diet mice were further divided into two groups, with one given control and another group given an HFHS diet. Maternal GDM in the absence of dietary stress in offspring resulted in the alteration of 89 proteins in the offspring’s ovaries. Canopy FGF signaling regulator 2 (CNPY2), deleted in azoospermia-associated protein 1 (DAZAP1), septin 7 (SEPT7), and serine/arginine-rich splicing factor 2 (SRSF2) were shown to be altered by GDM, adult dietary stress, or both. Overall, this study indicated the possible impact of GDM exposure in utero on the fertility and oocyte quality of offspring in later life.

## 7. Metabolomic Changes in Offspring after Exposure to Maternal Diabetes

Metabolomics addresses the quantitative profile of low-molecular-weight metabolites, such as amino acids, carbohydrates, lipids, or other compounds involved in a plethora of biological processes [50]. Since genetic and epigenetic regulation influences cellular homeostasis and leads to an altered metabolic output, the metabolome largely reflects environment–gene interactions and is a very sensitive measure of an organism’s physiological status [107]. To explore the metabolome, two distinct approaches are frequently utilized: the so-called untargeted approach, assessing the global profiles of all measurable analytes, and the targeted approach, focusing on a predefined set of compounds [108]. Even though the untargeted strategy offers an unbiased survey of molecules and can sometimes reveal novel, unexpected findings, many of the detected metabolites remain unannotated. However, a targeted approach, which is often used in hypothesis-driven studies and uses internal standards, allows accurate quantification of a variety of metabolites [109]. Mass spectrometry and nuclear magnetic resonance spectroscopy (NMR) are the two most commonly used analytical platforms in the field of metabolomics.

Given the constant transplacental supply of maternal metabolites towards the fetus and the fetuses’ own metabolism, metabolomics is a valuable tool to explore the effects of intrauterine exposure to elevated glucose at the molecular level. So far, GDM-related metabolomic studies are limited [110] and mainly refer to GDM-associated complications and their influence on the newborn cord blood metabolome (Table 5). Furthermore, it remains unclear if the metabolomic alterations observed in offspring born to diabetic women are due to an imbalanced maternal supply or alterations of the feto/placental metabolism or a combination of both.

To study the impact of GDM on cord plasma, Shokry et al. used LC-MS/MS to metabolically characterize samples from the PREOBE cohort [111]. Compared to non-GDM, elevated levels of the sum of hexoses were detected in both the maternal and cord blood, indicative of maternal hyperglycemia and increased glucose transport towards the fetus in GDM subjects. Importantly, uniquely in the cord blood but not in the maternal blood, free carnitine was significantly decreased. Furthermore, the same tendency was observed for acyl carnitines (AC), long-chain non-esterified fatty acids (NEFA), phospholipids (PL), specific Krebs cycle metabolites, and β-oxidation markers [112]. Moreover, also in the study by Dube et al., elevated cord blood glucose levels were found in newborns of GDM women, again reflecting the consequences of maternal hyperglycemia [113]. Interestingly, using mass spectrometry-based untargeted metabolomics, alterations of phospholipid levels during childhood, associated with GDM and persisting during adolescence, were also found in the EPOCH cohort [81]. This study further confirmed an association of the phospholipid metabolic pattern with increased adiposity, impaired insulin sensitivity, and altered adipocytokines across the adolescent transition among girls exposed to in utero GDM [114]. Likewise, Ott et al. aimed to investigate whether women with GDM and their offspring show similar metabolomic patterns eight years after birth. Intergenerationally correlated metabolites included carnitine (C0), glycerophospholipid (PC ae C34:3), two biogenic amines (taurine, creatinine), an amino acid (proline), and sphingolipid (SM-(OH) C14:1). The authors suggested a possible long-term programming effect of maternal GDM on metabolic health in children [115]. Conversely, even though Shokry et al. also found altered metabolites in the cord blood, associated with anthropometric changes in newborn children of mothers with GDM and obesity, these alterations were not detected longitudinally. As a conclusion, the study postulated a lack of predictive power of the cord blood metabolome for the later development of the children [116]. Lu et al. compared cord blood samples of newborns from GDM and from non-GDM women, using flow injection analysis–electrospray ionization–tandem mass spectrometry (FIA-ESI-MS/MS). The authors demonstrated that phosphatidylcholine acyl-alkyl C 32:1 and proline were associated with maternal GDM. Further statistical analysis showed that this association was independent of known GDM risk factors. The authors hypothesized that such an independent association might support the idea that the fetal metabolome may contribute to the development of maternal GDM [117]. Interestingly, Cetin et al. demonstrated that although proline (Pro), methionine (Met), isoleucine (Ile), alanine (Ala), leucine (Leu), and phenylalanine (Phe) were elevated in the umbilical vein blood from offspring born to women with GDM, these amino acids were unchanged in the maternal circulation. Even though ornithine concentration was increased in both the maternal and umbilical vein blood in GDM pregnancies, the levels in the umbilical vein and maternal blood were not significantly correlated. Strikingly, elevated fetal glutamate (Glu) and decreased glutamine (Gln) were also observed while they were unchanged in the maternal circulation. In this context, an increased hepatic Gln-to-Glu conversion, as a consequence of endocrine changes in the fetus, was suggested [118]. Pitchika et al. used UHPLC-MS and non-targeted metabolomics to examine fasting serum samples from the TEENDIAB [119] and BABYDIAB/BABYDIET [120] cohorts. The study included fasting serum samples from offspring born to mothers with type 1 diabetes and a control group of newborns born to non-diabetic mothers but with fathers or siblings with type 1 diabetes. With this study design, the authors aimed to elucidate to what extent health complications in offspring are due to parental genetic transmission or due to intrauterine exposure to hyperglycemia. The authors found that offspring of mothers with type 1 diabetes are more prone to worsening of the metabolic profile than offspring of fathers with type 1 diabetes. This provides evidence that in utero exposure to hyperglycemia has more influence on offspring than parental genetic transmission. Furthermore, increased levels of fasting glucose, insulin, and C-peptide were found in offspring of mothers with type 1 diabetes, but no significant associations between maternal type 1 diabetes and metabolite concentrations in offspring were observed. Overall, the authors proposed that maternal type 1 diabetes is associated with the offspring’s metabolic health, but this is unlikely to be caused by alterations in the offspring’s metabolome [121]. Lowe et al. used LC-MS/MS-based targeted metabolomics to elucidate the effect of maternal hyperglycemia on newborn cord blood from the HAPO cohort. They found that the maternal response to a glucose load, as reflected by maternal 1h glucose levels, was correlated with concentrations of 3-hydroxybutyrate and its carnitine ester, glycerol, and medium-chain carnitine ester in the cord blood [122]. Walejko et al. used ^1^H-NMR to study the effect of PGDM and GDM on serum metabolic changes in the cord blood at birth. The authors found that metabolites of the carbohydrate and choline metabolism were altered in the umbilical cord blood of newborns with both PGDM and GDM [123].

Peng et al. used liquid chromatography coupled to mass spectrometry to investigate the influences of GDM on the newborn meconium and urine metabolome. While in the urine, no significant differences between GDM and controls became apparent. Differences in the meconium metabolome pointed to significantly disrupted metabolic pathways, including lipid, amino acid, and purine metabolism. Moreover, the relationships between potential biomarkers and GDM risk were evaluated. Nine of them (argininosuccinic acid, methyladenosine, methylguanosine, aurodeoxycholic acid, glycocholic acid, hydroxyindoleacetylglycine, oxotrihydroxyleukotriene B4, tetrahydrodipicolinate, and DHAP (8:0)) showed the potential as markers for GDM-induced disorders [124]. In the study by Graca et al., using untargeted ultra-high-performance liquid chromatography-mass spectrometry (UPLC-MS)-based metabolomics, no significant alterations in the metabolome of amniotic fluid (AF) of GDM subjects, collected between gestational week 15 and 25, were detected [125]. It should be noted that AF from 10 weeks to 20 weeks is similar to fetal plasma, while the contribution of fetal urine is significant during the second half of the pregnancy [126].

Zhao et al. used ^1^H-NMR-based untargeted metabolomics to investigate the crosstalk between maternal gut microbiota and neonatal blood metabolome from pregnancies with GDM. The study showed that the maternal fecal metabolome and the matched neonatal blood metabolome could be separated along the vector of maternal hyperglycemia. A multi-Omics associated approach detected eight metabolites contributing to the close connection between the maternal fecal and the neonatal blood metabolome. Notably, in the feces, metabolites involved in biotin metabolism (lysine, putrescine, guanidinoacetate, and hexadecanedioate) were negatively correlated with maternal hyperglycemia [127].

Only a limited number of studies used animal models to investigate samples not accessible in a non-invasive way. Isganaitis et al. developed the “haploinsufficient for insulin receptor substrate-1 (IRS1-het)” mouse model. During pregnancy, despite normal body weights and plasma glucose levels, the mice are insulin-resistant and hyperinsulinemic, reflecting isolated maternal IR. Using this model, gas chromatography coupled to mass spectrometry was used to measure the liver lipids levels. The results revealed alterations of several lipid classes, notably in the fraction of the 16:1n7 family. However, at six months of age, the mice showed only a trend towards increased triglyceride species, while phospholipids were significantly reduced. The authors concluded that maternal IR could, even in the absence of hyperglycemia or obesity, promote metabolic perturbation in male offspring [128]. The liver metabolome was also targeted in a study by Pereira et al., where the offspring from rats with maternal obesity and GDM was compared to the offspring of lean dams. The offspring were further divided into two groups, with one given a high-fat and -sucrose diet (HFS) and the other a low-fat diet (LF). The analysis of the hepatic metabolome revealed increased diacylglycerol and reduced phosphatidylethanolamine levels in the offspring of GDM dams compared to offspring of lean dams. The authors concluded that GDM might be a driver of hepatic steatosis and insulin resistance in offspring [129]. Renner et al. developed a transgenic pig model expressing mutant insulin C93S in pancreatic β cells. The model mimics isolated maternal hyperglycemia without confounding obesity during pregnancy. Using LC/MS-based targeted metabolomics to analyze the offspring’s plasma metabolome, increased concentrations of lysine and α-aminoadipic acid, as well as phospholipids, were revealed. Moreover, the ratio of total and short-chain acylcarnitines to carnitine was elevated, indicating an increased import of fatty acids into mitochondria and an increased β-oxidation rate. Overall, it was shown that in this large animal model, even mild maternal hyperglycemia is associated with alterations in the plasma metabolome of the neonatal offspring [130].

**Table 5:** Summary of selected studies linking maternal diabetes with metabolome profiles of offspring.

Species	Maternal Characteristics	Bio-Specimen	Major Findings in Offspring	Reference
Human	GDM	Blood	Concentrations of lysine, putrescine, guanidinoacetate, and hexadecanedioate were negatively correlated with maternal hyperglycemia	[127]
Human	GDM	Cord Blood	Phosphatidylcholine acyl-alkyl C 32:1 and proline levels were associated with maternal GDM	[117]
Human	GDM	Blood	Association of the phospholipid metabolic pattern with higher adiposity, impaired insulin sensitivity and altered adipocytokines across the adolescent transition, among girls exposed to in utero GDM	[114]
Human	GDM	Blood	Intergenerational correlation of meta-bolites (carnitine, PC ae C34:3, taurine, creatinine, proline, SM-(OH) C14:1) between women with GDM and offspring 8 years after birth	[115]
Human	GDM	Cord blood	Elevated concentrations of Pro, Met, Ile, Leu, Ala and Phe; potentially, increased Gln-to-Glu conversion	[118]
Human	GDM and PGDM	Cord blood	Altered concentrations of metabolites of carbohydrate and choline metabolism	[123]
Human	GDM and overweight/obesity	Cord blood	Alteration of metabolites associated with anthropometric changes in newborn children, which were not detected longitudinally	[116]
Human	GDM and overweight/obesity	Cord blood	Elevated total hexoses; decreased levels of free carnitine, acyl carnitines, long-chain non-esterified fatty acids, phospholipids, specific Krebs cycle metabolites, and β-oxidation markers in cord blood but not in maternal blood	[112]
Human	Hyperglycemia	Cord blood	Concentrations of 3-hydroxybutyrate and its carnitine ester, glycerol and medium chain carnitine esters correlated with maternal 1h glucose levels	[122]
Human	GDM	Urine and meconium	No difference in urine; evidence for disrupted metabolic pathways, including lipid, amino acid, and purine metabolism from meconium analysis; argininosuccinic acid, methyladenosine, methylguanosine, aurodeoxycholic acid, glycocholic acid, hydroxyindoleacetylglycine, oxotrihydroxyleukotriene B4, tetrahydrodipicolinate, and DHAP (8:0) suggested as markers for GDM-induced disorders	[124]
Human	Type 1 diabetes	Serum	No significant associations between maternal type 1 diabetes and metabolite concentrations in offspring	[121]
Pig	Mutant insulin C93S causing hyperglycemia	Plasma	Increased concentrations of lysine, α-aminoadipic acid and phospholipids; biochemical evidence for an increased mitochondrial import of fatty acids for β-oxidation	[130]
Rat	GDM	Liver	Increased levels of diacylglycerol and reduced levels of phosphatidylethanolamine	[129]
Mouse	IR	Liver	Altered concentrations of the 16:1n7 lipid family; at 6 months of age a trend towards increased triglyceride species, while phospholipids were significantly reduced	[128]

## 8. Microbiomics and Nutriomics Studies Addressing GDM Effects on Offspring

Microbiomics is a fast-growing field, characterizing and quantifying the community of microorganisms found in a certain habitat [131]. The microbial community, also known as microbiota, consists of various microorganisms, such as bacteria, fungi, etc. The rapid development of high-throughput analytical technologies facilitated a detailed investigation of the microbiota with regards to their genetic and functional divergence and caused paradigm shifts in our understanding of their roles in health and disease [132]. Especially the sequence analysis of small subunit ribosomal RNA (16S rRNA) genes as well as shotgun metagenomics sequencing, in which total DNA is sequenced, are the leading methods utilized for microbiome profiling. Both approaches allow an accurate quantitative determination of microorganisms and their taxa, which can be correlated with disease or other phenotypes of interest [50].

At first, the fetal gut was thought to be sterile at birth. However, recent studies indicate that colonization of the gastrointestinal tract may already occur during fetal development and could also be influenced by maternal environmental exposures and by the mode of birth (caesarean section or vaginal) [133]. So far, there are only a few studies investigating the influence of maternal diabetes on offspring microbiota. Furthermore, studies are often confounded by varying perinatal conditions known to affect the microbiota colonization of offspring. This prevents a clear distinction of the maternal diabetes effects from those of concomitant diseases. Despite this, it seems that specific taxa associated with maternal diabetes can be transmitted to the offspring, which differentiates their microbiota from the offspring of non-diabetic mothers. A comprehensive review of studies investigating the influence of maternal GDM on the neonatal microbiome was recently published [134]. Therefore, here we summarize only some major findings from the studies shown in Table 6. For instance, Ponzo et al. compared the microbiota of offspring born to GDM mothers versus normoglycemic mothers by 16S amplicon-based sequencing of fecal samples collected during the first week of life. The microbiota of infants from GDM women showed a lower complexity and higher inter-individual variability. Furthermore, the relative abundance of pro-inflammatory taxa, in particular *Escherichia* and *Parabacteroides*, was increased [135]. Similarly, Soderborg et al. investigated the gut microbiota from 2-week old neonates born to normal-weight or overweight/obese mothers with or without GDM and found evidence that GDM alone or together with maternal overweight/obesity influences the infant microbiota in a way that sets the stage for the future risk of inflammatory and metabolic diseases [136]. Furthermore, Hu et al. explored the diversity of the meconium microbiome to determine if it is affected by maternal diabetes (pre-pregnancy type 2 diabetes and GDM). The authors concluded that the meconium microbiome of infants born to mothers with diabetes is enriched with similar bacterial taxa as those reported in the fecal microbiome of adult diabetic patients [137]. This finding might indicate a non-genetic risk of transmission of diabetes (i.e., through the microbiome). Therefore, it is interesting if similar findings are observed in longitudinal studies exploring the effect of maternal diabetes on the offspring microbiome postpartum. Using 16S rRNA gene amplicon sequencing, Crusell et al. compared feces from children of mothers with and without GDM. The samples were collected during the first week of life and later at an average age of 9 months. The results indicate that differences in glycemic control during late pregnancy are linked to relatively modest variations in the gut microbiota composition of offspring during the first week of life and nine months after birth. In addition, similar to the above-mentioned study by Ponzo et al. and the study by Su et al. [138], Crusell et al. found a lower richness of the gut microbiota in GDM neonates compared with neonates born to mothers without GDM. The authors also referred to the previous reports [139], where high-microbial richness was associated with metabolically more advantageous phenotypes (i.e., lower BMI, and higher insulin sensitivity, etc.). Furthermore, the authors found species in the gut microbiota that was similar to the microbiota observed in childhood obesity and in adults with type 2 diabetes [140]. In a longitudinal study by Hasan et al., stool samples from offspring of GDM and non-GDM mothers collected five years postpartum were analyzed for microbiome profiling. Interestingly, the *Anaerotruncus* genus was more abundant in the offspring of GDM mothers [141]. Of note, *Anaerotruncus* was also found to be enriched in the gut microbiota of GDM women even after the adjustment for pre-pregnancy BMI [142]. This may be one of the examples of an intergenerational concordance of microbial variation in mother–offspring pairs. Nevertheless, whether an increase in *Anaerotruncus* in the offspring microbiome is a risk factor for future metabolic disorders has to be elucidated in further studies.

Nutriomics is formed by the combination of nutritional science and different Omics techniques, resulting in disciplines such as nutrigenomics, and nutriproteomics, etc. Taking into account the complexity of the human body and its interplay with food, it is conceivable that holistic molecular analyses of food–body interactions (i.e., nutri-omics) are essential to understanding the effects of nutrients [143]. Several studies have pointed to diet–gene interactions affecting glucose metabolism and being linked to diabetes (reviewed in [144]). Studying dietary interventions and personalized nutrition could reveal certain diet types, which could help prevent adverse fetal outcomes caused by maternal diabetes.

Studies investigating combinatorial effects of maternal diabetes, the type of offspring diet, and the influence of both on offspring health are limited (Table 7). Results from the longitudinal, large-scale cohort study by Chu Tint et al. suggested that a high placental inositol content may reduce the pro-adipogenic effects of maternal glycemia, resulting in lower birth weight and reduced adiposity of offspring. The authors used the longitudinal data from the Growing Up in Singapore Towards healthy Outcomes (GUSTO) cohort [145]). Maternal FPG and 2h plasma glucose (2 hPG) were obtained in pregnant women by a 75 g oral glucose tolerance test at around 26 weeks of gestation. Placental inositol was quantified by liquid chromatography-mass spectrometry. Interestingly, maternal glycemia and fetal birth weight (or abdominal adiposity) correlated positively only in the case of low, but not high, placental inositol content. The authors emphasized the anti-adipogenic effect of inositol during maternal hyperglycemia and pointed to the potential benefits of prenatal inositol supplementation [39]. Interestingly, a study by Pereira et al. found that metabolic disturbances observed in the liver from offspring of gestational diabetic dams were even worsened after the challenges of a high-fat diet. A low-fat diet did not show protective effects against these metabolic phenotypes (obesity, hepatic steatosis, and insulin resistance) [129] (see the chapter: Fetal metabolomic changes after exposure to maternal diabetes).

## 9. Conclusions

Diabetes is a serious health concern for pregnant women and their offspring. In this article, we have reviewed the contributions of Omics technologies in studying the effects of maternal diabetes on offspring health. The transformation of complex and heterogeneous Omics data into biological meaning is a daunting task. In this regard, careful interpretation of results, especially from studies with limited numbers of samples, is necessary. Overall, there is increasing evidence pointing to molecular disturbances in offspring exposed in utero to maternal diabetes. Apparently, many characteristics that are based on observational cohort studies are reflected on molecular levels in Omics studies, demonstrating that maternal diabetes influences the cellular and organ systems of the offspring. This includes the predisposition of offspring to future obesity triggered by elevated maternal glucose levels. Other long-term offspring health complications include cardiovascular and neurodevelopmental disorders, as supported by Omics studies. It should be noted that the majority of the Omics studies reviewed here were performed at birth, and the knowledge about molecular derangements during earlier stages of embryo development is lacking. More Omics experiments using animal models would be valuable to close this knowledge gap and to reveal the most susceptible windows during development when alterations due to maternal diabetes manifest. Additional research is also warranted to determine if Omics data will result in new biomarkers for the diagnosis, treatment, and prevention of offspring health problems related to maternal diabetes.

## Figures and Tables

**Table 1:** Maternal and fetal/offspring risks associated with maternal diabetes [19,22,23,24,25,26,27,28].

Maternal	Fetal/Offspring
Pre-eclampsia	Intrauterine death
Cesarean section	Congenital malformations
Labor complications	Macrosomia
Pre-term delivery	Polycythemia and hyperbilirubinemia
Postpartum hemorrhage	Respiratory distress syndrome
Recurrent GDM	Insulin resistance Metabolic syndrome
Type 2 diabetes	Type 2 diabetes
Complications of type 2 diabetes (cardiovascular disease, nephorpathy, neuropathy, retinopathy)	Complications of type 2 diabetes (cardiovascular disease, nephropathy, neuropathy, retinopathy)
weight gain/obesity	Weight gain/obesity

**Table 4:** Summary of selected studies linking maternal diabetes with proteome profiles of offspring.

Species	Maternal Characteristics	Bio-Specimen	Major Findings in Offspring	Reference
Human	GDM, type 1 diabetes, type 2 diabetes	Cord blood	Altered abundance of APOM, CP, PLG, AGT, KNG1, APOA1, ORM2, TF, HRG, APOD, LUM; processes such as inflammation, extracellular matrix remodeling, lipid metabolism, etc. mainly affected	[102]
Human	GDM	Umbilical venous plasma	Altered abundance of CEPT and APOM; FSH as upstream regulator of the differentially abundant proteins	[104]
Human	GDM	Umbilical venous plasma	Altered abundance of PLTP and LCAT (related to abnormal glucose and lipid metabolism) and ARHGEF11 (known to influence embryo development)	[103]
Mouse	Diet-induced diabetes	Ovaries	Altered abundance of CNPY2, DAZAP1, SEPT7, and SRSF2; potential impact on fertility and oocyte quality of offspring in later life	[106]

**Table 6:** Summary of selected human studies linking maternal diabetes with microbiome profiles of offspring.

Maternal Characteristics	Bio-Specimen	Major Findings in Offspring	Reference
GDM	Feces	GDM alone or together with maternal overweight/obesity influences infant microbiota in a way that set the stage for future risks of inflammatory and metabolic disease	[136]
GDM	Feces	Glycemic regulation in late pregnancy is linked with relatively modest variation in the gut microbiota composition of the offspring at age 1 week and 9 months; lower richness of the gut microbiota in GDM neonates compared with neonates born to mothers without GDM	[140]
GDM	Feces	Increased relative abundance of pro-inflammatory taxa, in particular *Escherichia* and *Parabacteroides*	[135]
GDM	Feces	Increased abundance of *Anaerotruncus* genus	[141]
Type 2 PGDM GDM	Meconium	Enrichment of the meconium microbiome for the same bacterial taxa as reported in the fecal microbiome of adult diabetic patients	[137]

**Table 7:** Summary of selected studies investigating combinatorial effect of maternal diabetes and offspring nutrition on offspring health.

Species	Maternal Characteristics	Major Findings in Offspring	Reference
Human	Hyperglycemia	Positive correlation of maternal glycemia and fetal birth weight/abdominal adiposity in the case of low, but not high, placental inositol content	[39]
Rat	GDM	Metabolic disturbances in liver of offspring from gestational diabetic dams worsened upon a high-fat diet; no protective effect of a low-fat diet against metabolic changes (obesity, hepatic steatosis, insulin resistance)	[129]

## Data Availability

Not applicable.

## Abbreviations

16S rRNA	16S ribosomal RNA
2 hPG	2-h plasma glucose
Ala	Alanine
ABCA1	ATP-binding cassette transporter A1
AC	Acyl-carnitine
ADIPOQ	Adiponectin
AF	Amniotic fluid
AGT	Angiotensinogen
Ahsp	α haemoglobin stabilizing protein
AMPK	AMP-activated protein kinase
APOA1	Apolipoprotein A-I
APOD	Apolipoprotein D
APOM	Apolipoprotein M
ARHGEF11	Rho guanine nucleotide exchange factor 11
BMI	Body mass index
CEPT	Cholesteryl ester transfer protein
ChiP	Chromatin immunoprecipitation assay
CNYP2	Canopy FGF signaling regulator 2
CP	Ceruloplasmin
CpG	Cytosine-guanine dinucleotide
CVD	Cardiovascular disorder
CYP2E1	Cytochrome P450 Family 2 Subfamily E Member 1
DAZAP1	Azoospermia Associated Protein 1
DEG	Differentially expressed genes
DNA	Deoxyribonucleic acid
EFSOCH	The Exeter Family Study of Childhood Health
EGR1	Early growth response 1
ELISA	Enzyme-linked immunosorbent assay
EPOCH	Exploring Perinatal Outcomes among Children
FIA-ESI-MS/MS	Flow injection analysis-electrospray ionization-tandem mass spectrometry
FOXO1	Forkhead box protein O1
FPG	Fasting plasma glucose
FSH	Follicle-stimulating hormone
GDM	Gestational diabetes mellitus
Gln	Glutamine
Glu	Glutamate
GWAS	Genome-wide association study
HAPO	The Hyperglycemia and Adverse Pregnancy Outcome
HFHS	High fat, high sucrose diet
HFS	High fat diet and sucrose diet
HIF1	Hypoxia-inducible factor 1
HRG	Histidine-rich glycoprotein
HUVEC	Human umbilical vein endothelial cells
Ile	Isoleucine
IPA	Ingenuity Pathway Analysis
IR	Insulin resistance
IRS1-het	Haploinsufficient for insulin receptor substrate-1
Kel	Kell metallo-endopeptidase
KNG1	Kinogen-1
LCAT	Lecithin–cholesterol acyltransferase
LC-MS/MS	Liquid Chromatography with tandem Mass Spectrometry
LDL	Low-density lipoprotein
Leu	Leucine
LF	Low fat diet
LGA	Large for gestational age
LPL	Lipoprotein lipase
LUM	Lumican
LV	Left ventricle
MEST	Mesoderm Specific Transcript
Met	Methionine
MHC	Major Histocompatibility Complex
MIA	Maternal immune activation
mRNA	Messenger RNA
NEFA	Non-esterified fatty acid
NGS	Next generation sequencing
NMR	Nuclear magnetic resonance
OGTT	Oral glucose tolerance test
OR2L13	Olfactory Receptor Family 2 Subfamily L Member 13
ORM2	α-1-acid glycoprotein 2
PACE	Pregnancy and Childhood epigenetics consortium
PCK1	Phosphoenolpyruvate Carboxykinase 1
PGDM	Pregestational diabetes mellitus
Phe	Phenylalanine
PL	Phospholipid
PLG	Plasminogen
PLTP	Phospholipid transfer protein
PPAR	Peroxisome proliferator-activated receptor
PPARGC1α	Peroxisome proliferator-activated receptor-γ, co-activator 1, α
Pro	Proline
RDH16	Retinol Dehydrogenase 16
RNA	Ribonucleic acid
SETP7	Septin 7
SH3PXD2A	SH3 And PX Domains 2A
SLC6A4	Serotonin transporter gene
SNP	Single nucleotide polymorphism
SOD2	Superoxide dismutase 2
SRSF2	Serine and Arginine Rich Splicing Factor 2
STZ	Streptozotocin
TF	Serotransferrin
UHPLC-MS	Ultra-high-performance liquid chromatography Mass spectrometry
Vegfa	Vascular endothelial growth factor A
WB	Western blot
WGCNA	Weighted Correlation Network Analysis
